# WHO ARE THE PORTUGUESE WHO REACH 100 YEARS OF AGE? A DEMOGRAPHIC ANALYSIS USING 2021 CENSUS DATA

**DOI:** 10.1093/geroni/igad104.2330

**Published:** 2023-12-21

**Authors:** Oscar Ribeiro, Lia Araujo, Laetitia Teixeira

**Affiliations:** University of Aveiro, Aveiro, Aveiro, Portugal; Cintesis, Porto, Porto, Portugal; School of Medicine and Biomedical Sciences, University of Porto, Porto, Porto, Portugal

## Abstract

Portugal is considered one of the oldest countries of Europe as a conjugation of low birth rates and high life expectancy. The age group of 100+ is one of the fastest growing, with 270 centenarians in 1990 to 1961 centenarians in 2020 (Teixeira et al., 2020). In this study we present the profile of centenarians based on real data of the last Portuguese Census 2021. There are 2801 centenarians, showing a significant increase in relation to the previous censuses (589 centenarians in 2001, and 1526 centenarians in 2011). Most centenarians are women (82.0%), widowed (84.6%), attended school (61.0%), have social pension as main income source (96.3%), are catholic (93.9%) and were born in Portugal (98.6%). The average number of reported difficulties in vision, hearing, mobility, self-care, memory/concentration, and communication is of 5.12, meaning that the current cohort of centenarians face high constrains in their daily living and most probably depend on any sort of care support. Overall, Portugal follows the longevity phenomenon of a growing number of centenarians as verified in USA, Japan, and other European countries (United Nations, 2022). Profiling centenarians’ main characteristics with reliable data is a starting point for outlining the specific social, medical, and financial needs of those reaching very advanced ages, and for defining government policies and age-sensitive interventions.

